# Minimizing nutrient losses to freshwater from pasture-based dairy systems

**DOI:** 10.3168/jdsc.2025-0934

**Published:** 2026-05-01

**Authors:** R.W. McDowell, K.A. Macintosh, J.R. Roche, O. Fenton, L. Delaby

**Affiliations:** 1 Faculty of Agriculture and Life Sciences, Lincoln University, Lincoln, New Zealand 7608; 2 New Zealand Institute for Bioeconomy Sciences, Lincoln University, Christchurch, New Zealand 7608; 3 School of Geography and Environmental Sciences, Ulster University, Coleraine, Northern Ireland BT52 1SA; 4 School of Biological Sciences, University of Auckland, Auckland, New Zealand 1142; 5 Teagasc, Johnstown Castle, Environment Research Centre, Wexford, Ireland Y35 TC97; 6 INRAE, L'Institut Agro, UMR Physiologie, Environnement et Génétique pour l'Animal et les Systèmes d'Elevage, 35590 Saint Gilles, France

## Abstract

Pasture-based dairy systems have expanded in regions with suitable climates, often driven by consumer demand for perceived environmental, animal welfare, and nutritional advantages. Expansion and intensification of such systems have increased nitrogen (N) and phosphorus (P) losses to water, contributing to eutrophication and groundwater contamination. To support sustainable milk production and reduce environmental impact, it is necessary to understand the transport pathways of nutrient losses and the actions that will mitigate the same. Nutrient losses arise from interactions between sources, mainly animal excreta, imported feed and fertilizer, soil, and pasture, and transport pathways such as runoff (surface or near surface in tile drainage) and leaching. Whereas urine patches dominate N loss in pasture-based systems, through nitrate (NO_3_-N) leaching, dung and soil are key P sources, especially via surface runoff under wet or compacted soil conditions. Actions to mitigate losses must be tailored to N or P and range from farm system management practices, such as improved nutrient management, manure handling, and strategic grazing or housing/off-paddock infrastructure, to edge-of-field mitigations like appropriate placements of wetlands, riparian buffers, denitrification beds, and detainment bunds. Targeting actions to critical source areas can greatly enhance their cost-effectiveness. Successful implementation of actions depends on aligning environmental benefits with farm profitability, supported by policy instruments that blend voluntary and regulatory approaches.

## Highlights

• Nutrient losses to freshwater depend on sources and transport pathways.
• Urine patches dominate nitrate-nitrogen leaching in pasture-based systems.
• Dung and soil are primary phosphorus sources lost via surface runoff.
• Actions must be nutrient specific and range from farm system to edge-of-field.
• Targeting actions to critical source areas increases cost-effectiveness.

## Graphical Abstract

Nutrient losses arise from the interaction of nutrient sources like animal excreta, imported feed, fertilizer, and soil, with transport pathways such as surface runoff and subsurface flow. Urine patches dominate nitrate nitrogen leaching, especially when deposited on free-draining soils before wet periods, while dung, effluent, soil, and sediment are key sources of phosphorus loss via runoff. Mitigating losses includes actions such as nutrient budgeting, effluent management, strategic grazing, and off-paddock infrastructure, and edge-of-field interventions such as wetlands, riparian buffers, and denitrification beds. Targeting actions to critical source areas of nutrient loss can increase their cost-effectiveness and support measurable improvements in freshwater quality.

**Figure:**
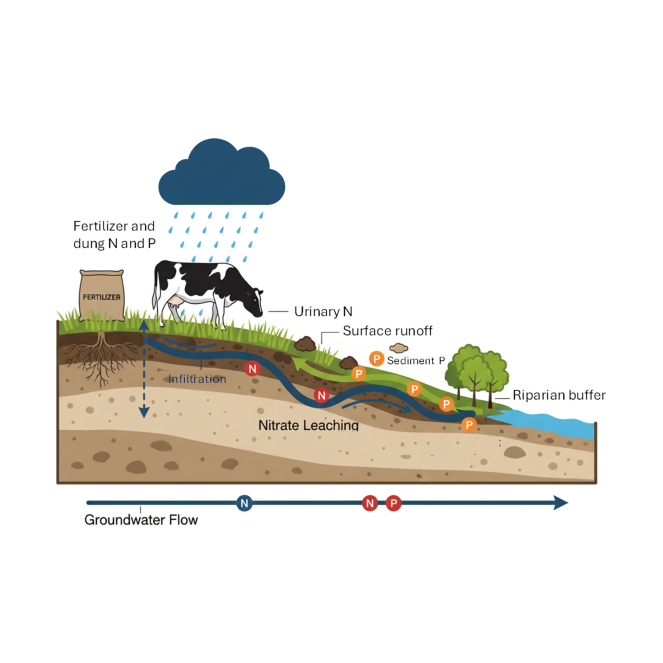

Pasture-based dairy systems contribute approximately 10% to 15% of global milk production (Dillon and Fenelon, 2018; Shalloo et al., 2018). They are defined as systems where cows obtain most of their feed intake on a fresh-weight basis from grazed pasture during lactation. Cows therefore consume <20% of their diet when lactating as DM from supplementary feed (1 t DM/cow per yr) and spend most of the lactating period or more outdoors grazing pasture. Pasture-based dairying has intensified in Europe (especially France, the United Kingdom, and Ireland), southeastern Australia, New Zealand, pockets of North and South America, and South Africa, where there is a favorable climate (Fetzel et al., 2017; Hennessy et al., 2020). In addition to economic efficiency, expansion has been driven by perceived environmental and animal welfare benefits. However, in a recent review, McDowell et al. (2022) found insufficient evidence to support the assertion that grazed dairy systems have lower nutrient losses compared with housed systems. Instead, evidence indicates that hybrid systems, where cows graze <8 mo per year and are housed for the remainder, could reduce nutrient (nitrogen [N] and phosphorus [P]) losses (Figure 1). This is achieved through the storage of excreta during winter for later land application as an organic fertilizer source, while also mitigating soil damage caused by animals grazing in inclement weather and on waterlogged soils.

**Figure 1:**
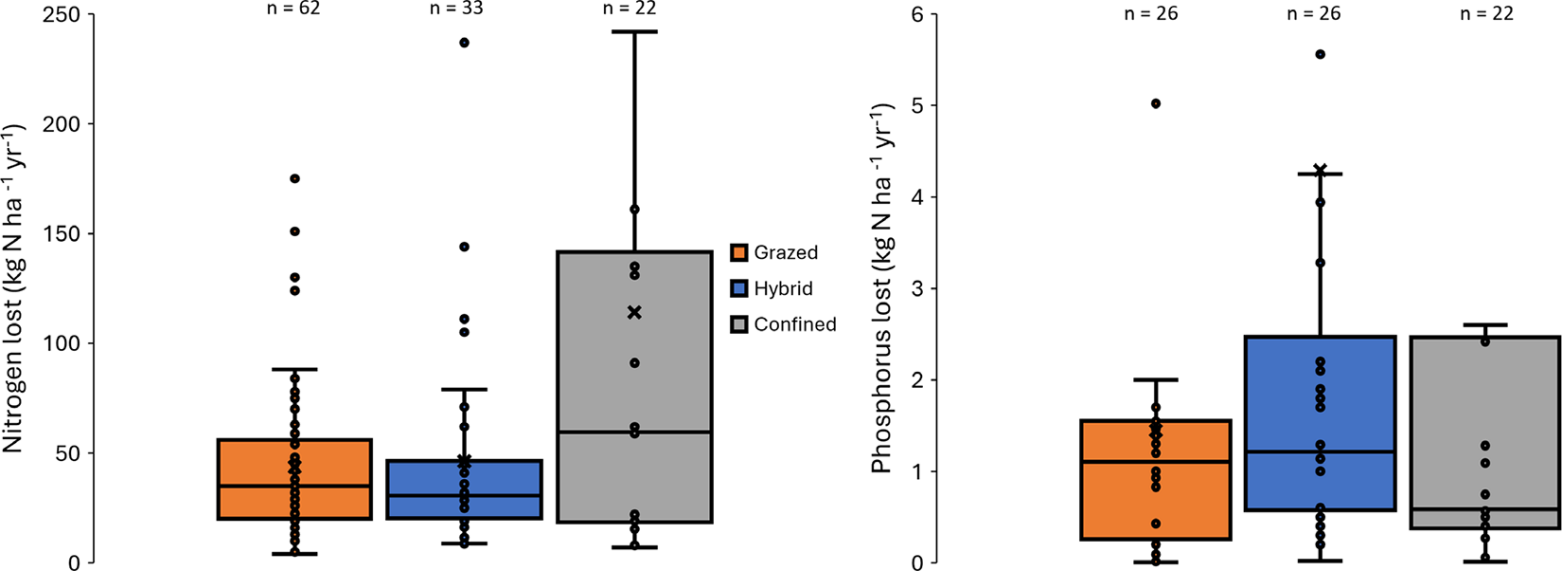
Data comparing nitrogen and phosphorus losses from grazed, confined, and hybrid dairy farm systems, globally. Data from McDowell et al. (2022). Boxes span the interquartile range (25th–75th percentiles), with the horizontal line indicating the median; whiskers extend to the 5th and 95th percentiles, and dots represent outliers.

Producing milk from pasture, like most other agricultural food items, has an associated environmental footprint and can lead to increased losses of nutrients from land to water (Clay et al., 2020). Enriched nutrient concentrations can stimulate excessive algal growth in surface waters (McDowell et al., 2025b) and, in the case of NO_3_-N, compromise groundwater potability (Morgenstern and Daughney, 2012). As scientific understanding evolves, it is timely to re-examine and summarize the key factors influencing nutrient loss rates, their sources and transport pathways, and the actions and policies to mitigate those losses.

An important factor contributing to nutrient losses is the inherent complexity of pasture-based systems. Farmers must balance pasture production and quality, feed demand, and plant nutrient uptake under variable climatic conditions, creating multiple points in time and space where nutrients can be lost. For instance, in New Zealand, national modeling suggests that N losses from dairy farms in 2015 would have been about 40% higher had farmers not implemented actions to apply effluent to land, fence off streams, and improve fertilizer use and irrigation efficiency (Monaghan et al., 2021).

Nutrient losses from grazed dairy systems can be conceptualized as the interaction between nutrient sources (e.g., excreta, fertilizer, soil, and feed inputs) and transport pathways (surface runoff, subsurface flow, and leaching), with their relative importance being nutrient specific (Heathwaite et al., 2000). Of note, fertilizer is a minor direct source of N and P loss (<10% of total losses) when applied at appropriate rates and times. However, the potential for loss increases when fertilizer use greatly exceeds plant demand, and applications occur before or during heavy rainfall, or coincide with periods of limited plant uptake such as during cool or waterlogged conditions. An unintended consequence of lowering fertilizer application rates to reduce nutrient losses can occur by increasing imported feed supply either as pasture or supplemental feed. Although this can support greater milk production and higher animal numbers, it may also increase nutrient cycling within the soil-plant system and the quantity of nutrients returned to soil via urine and dung, thus increasing losses.

For N, approximately 70% to 80% of ingested N in pasture-fed cows is excreted, the majority in urine. Urine patches can contain 200 to 2,000 kg N/ha (Selbie et al., 2015), far exceeding plant uptake capacity. Following deposition, N is rapidly converted to water-soluble NO_3_-N and becomes vulnerable to leaching through the soil profile when drainage occurs. Losses are greatest when urine is deposited on free draining soils before rainfall events, typically in late autumn or early winter, when plant uptake is low and drainage volumes are high.

Given the dominance of urine as a N source, livestock numbers are an important driver, although variation in feed inputs and animal productivity means stocking rate alone can be a coarse indicator of N loss rate. Metrics such as lactating cow grazing days per hectare or farm-gate N surplus often provide more robust indicators of leaching risk (Vérité and Delaby, 2000; Chapman et al., 2018). Nevertheless, relationships between stocking rate and N loss are not linear. For example, higher stocking rates without supplementary feed imports can reduce NO_3_-N leaching per hectare due to greater N export in product and more even distribution of excreta (Roche et al., 2016).

This relationship is important for management. Some have suggested providing low-N supplementary feed in autumn and winter to reduce dietary crude protein and, consequently, urinary-N concentration, lowers NO_3_-N leaching (Wilkinson and Waldron, 2017). However, this strategy increases imported feed and maintaining profitability typically requires an increase in stocking rate to ensure pasture utilization is maintained (Neal and Roche, 2019). As a result, total N load during autumn increases, offsetting any benefit from the lower-N diet, and lactating cows require higher N intake than nonlactating cows, resulting in higher NO_3_-N leaching (Ledgard et al., 2006). This illustrates that decision-making is not straightforward.

Most P is excreted in feces and deposited directly onto pasture or soil (or both) or is collected as part of cleaning the milking parlor and stored as farm dairy shed effluent for land application. The potential for P loss to surface runoff from dung declines exponentially with time as fecal material dries. Unlike dairy farms that house livestock and must store dung as manure or slurry, in pasture-based systems, the milking parlor effluent is more dilute (<1% solids compared with 30%–40% solids for manure/slurry), which can lead to an increased P loss risk (and organic-N) when effluent applied exceeds plant nutrient uptake or when soils are saturated.

Soil is often a dominant (>50%) source of P loss from dairy pastures (McDowell et al., 2007). Moderate to high P fertilizer (35–70 kg P/ha) is used to support a high pasture legume content by maintaining recommended agronomic levels of soil test P. Applied P (fertilizer or excreta) is retained by sorption to aluminum and iron oxides or precipitated as calcium or magnesium phosphates. The risk of P loss in dissolved (<0.45 μm) form increases exponentially when soil test P is above the agronomic optimum (e.g., 20–30 mg Olsen P/kg), but may also occur at low soil test P if the soil contains little aluminum or iron (e.g., organic soils; McDowell et al., 2008). For N, soil represents a minor source of N loss, often only made available through cultivation, where soil disturbance by plowing stimulates the biological turnover of N in organic matter into transportable dissolved organic and NO_3_-N forms (Monaghan et al., 2007). Biological activity may also increase and mineralize N when soils become wet after a period of drought. There is a need to integrate legacy N storage dynamics and time lags into policy and practice as these concepts invalidate steady-state models often used in policy, and quantifying soil N storage would align better with P and carbon cycling research (Ascott et al., 2021).

Transport pathways for N and P occur via both surface runoff and subsurface flow, although the dominant nutrient form and proportion lost can differ between pathways. Surface runoff (overland flow) occurs via infiltration—excess during high-intensity rainfall, or saturation—excess when soils are already wet, with the former often promoting greater particulate losses (Kleinman et al., 2006). Climate change is expected to intensify these processes, with more extreme rainfall events increasing infiltration—excess runoff in some regions, and wetter winters increasing saturation—excess runoff in others. Grazing on wet soils can disproportionately contribute to nutrient losses due to increased soil damage, compaction, and limited plant uptake.

Actions to mitigate the loss of nutrients to freshwater can be grouped into farm system management practices and edge-of-field mitigations. Farm system management practices include judicious fertilizer use, the recycling of livestock excreta, grazing management, and management of lactation length, or use of housing or off-paddock infrastructure (Table 1). When effectively implemented, these practices can reduce nutrient losses and improve production. Applying fertilizers based on soil testing and plant requirements (including growing conditions) ensures nutrients are used effectively while lowering nutrient losses and input costs (Macintosh et al., 2019). Similarly, treating livestock excreta not as waste but as a nutrient source can lower fertilizer reliance (Liu et al., 2018), whereas not overgrazing can avoid soil compaction, erosion, and impaired pasture productivity. Moderating the use of imported feeds or DIM (or both) and providing housing or off-paddock infrastructure (e.g., for use during inclement weather) can reduce pressure on saturated soils, limit surface runoff (from fields and roadways) and near-surface runoff (tile drainage), improve animal welfare, and enable manure collection, storage, and delaying manure applications to when soils are drier.

**Table 1:** Performance characteristics of common actions to lower N and P loss from pasture-based dairy systems (adapted from a full list available in McDowell et al., 2025a)

Action	Effectiveness^1^ (%)	Relative cost^2^ ($/ha per yr)	Co-benefits	Factors limiting uptake
Fertilizer (e.g., nutrient management plan/budget, 4R's,^3^ slow-release forms)	Low [N] Mod [P]	Low [N; P]	Decrease emissions of greenhouse gases (e.g., N_2_O)	Time and expertise to prepare and carry out
Farm dairy shed effluent (e.g., greater effluent storage, low rate application, greater land application area)	Mod [N; P]	Mod [N] Low [P]	If more N and P is adsorbed in the soil, it will reduce fertilizer requirements.	Requirement for storage is dictated by local climate and if too wet, may make practice very expensive or to reduce shed water use. Availability of suitable land. Increased labor requirements depending on method of application.
Grazing management, less supplementary feed use, particularly in autumn, and housing infrastructure (e.g., reduced DIM, restricted grazing, use of off-paddock infrastructure)	High [N] Mod [P]	High [N; P]	Decreased soil and pasture damage and N_2_O emissions	Reduced milk production/cow through fewer DIM and high capital and operational costs. Must be accompanied by housing/off-paddock infrastructure that has no connection to a waterway (e.g., runoff/effluent is captured) and adequate effluent storage.
Wetland (e.g., natural, constructed)	High [N] Low [P]	Mod [N] High [P]	Flood attenuation, landscape aesthetics, wildlife habitat, and biodiversity	No suitable areas on farm; cost of establishment
Riparian planting/buffer strips	Mod [N; P]	High [P]	Stream bank stabilization. Provides stream shading, improved aquatic habitat, carbon supply to streams, landscape aesthetics, and recreational and cultural benefits	Land adjacent to stream may not be available or suitable for a buffer strip. Cannot intercept artificial drainage. Takes land out of production. Will require ongoing maintenance (e.g., weed control).
Denitrification beds	High [N]	High [N]	Could be integrated to support removal of dissolved P.	May require resource consent; cost of establishment
Detainment bunds (or retention ponds)	Mod [P]	High [P]	Potential to buffer storm events and therefore potential downstream flooding	May require resource consent; cost of establishment

1 Kilograms of nutrient retained or immobilized and categorized into low, moderate (Mod), and high = 0%–33%, 34%–66%, and 67%–100%, respectively.

2 Cost breakdowns for each quarter were (low, medium, and high): N (<100, 101–366, and >366 $/ha per yr); P (<111, 112–476, and >476 $/ha per yr). Cost estimated in 2020.

3 Refers to right rate, right time, right place, and right product.

Edge-of-field actions include wetlands (either natural or constructed), fencing stock out of waterbodies, restoring riparian margins through planting (Goeller et al., 2020) and the use of denitrification beds and detainment bunds. The primary objective of wetlands on agricultural land is to remove NO_3_-N from surface water (Audet et al., 2020), although they can also act as a sink for both sediment and particulate P. Wetlands operate by slowing water flow and providing conditions conducive to denitrification. Fencing livestock out of waterbodies prevents direct fecal contamination and bank erosion via slumping (Muirhead, 2019). Restoring riparian planting along waterways can stabilize streambanks, trap sediment, and intercept surface runoff before it reaches freshwater systems (Stutter et al., 2025). Denitrification beds use carbon (C)-rich woodchips to intercept drain flow and convert NO_3_-N to N gas, which is lost to the atmosphere (Burbery et al., 2022). Detainment bunds (or retention ponds) slow the flow of water during storm events and allow coarse-sized sediment and associated N and P to settle out (Levine et al., 2021).

Edge-of-field actions are not a substitute for farm system management practice change. Instead, they provide an alternative line of defense and can deliver co-benefits, including habitat creation, increased biodiversity, and C-sequestration (Ó hUallacháin et al., 2025). To optimize the performance of mitigation actions (e.g., Table 1), land managers should consider their suitability and longevity and maximize their cost-effectiveness by targeting them, where possible, to critical source areas (i.e., small areas of a field or farm that account for most nutrient losses). Targeting critical source areas can increase the cost-effectiveness of actions for P up to 7-fold compared with an untargeted approach (McDowell, 2014).

Many drivers influence the implementation rate of actions to mitigate the loss of nutrients from grazed dairy land. They include extension advice, voluntary sector-led initiatives, the use of farm environment plans, agri-environment schemes, policy, and regulation (Wuepper et al., 2024). Farmers strive to minimize costs and maximize profitability; mitigation actions that align with those outcomes are, therefore, more likely to be implemented voluntarily (McDowell et al., 2016). However, this also means that environmentally beneficial actions with higher capital costs may require regulation or financial incentives to achieve widespread adoption in the absence of subsidies. Voluntary schemes often rely on farmer goodwill, leadership from industry or sector bodies, and catchment groups. In contrast, regulatory regimens impose minimum standards and ensure compliance across the agricultural production sector, which can be essential when voluntary measures alone are insufficient or are too slow to generate change (McDowell et al., 2016).

New Zealand and Europe provide examples of catchments dominated by dairy pastures that have used a mix of voluntary and regulatory approaches to reducing nutrient loss. For example, in New Zealand, voluntary dairy sector initiatives such as “Water Accords” (Dairy Environment Leadership Group, 2013; Ministry for Primary Industries, 2013) have been supported by regulations (e.g., National Policy Statement for Freshwater Management; Ministry for the Environment, 2025) to help drive the uptake of actions (Macintosh et al., 2025). Collaboration between primary industries and the government has resulted in farm environment plans being proposed for all farmers. These plans identify and implement (through auditing) an optimized array of farm-scale and catchment-specific actions to reduce nutrient losses (Ministry for the Environment, 2021). The implementation of actions in these plans has been shown to improve water quality at a catchment scale (McDowell et al., 2023), while maintaining farmer engagement and a degree of autonomy on how water quality is improved.

In Europe, agricultural production has long been financially supported through subsidies and agri-environmental schemes through the European Union's Common Agricultural Policy, the Water Framework Directive, the Nitrates Directive, and the European Commission Farm to Fork Strategy (Wuepper et al., 2024). These schemes are also used to provide financial support to farmers who voluntarily implement actions that go beyond mandatory environmental standards. Decreases in nutrient concentrations have largely been attributed to reductions from point sources (Cooper and Hiscock, 2023) with few improvements seen in large catchments (Wiering et al., 2020). However, smaller catchments (e.g., <100 km^2^) can see a quicker response to the implementation of actions, owing to a larger agricultural signal and short lag times (Melland et al., 2018).

Taken together, this mini-review shows that nutrient losses from pasture-based dairy systems are driven less by farm system type and more by how nutrients are efficiently managed across space and time within complex farm environments and catchments. The effective implementation of mitigation actions should therefore manage the interactions between nutrient sources and transport pathways, particularly by targeting high-risk loss periods and critical source areas. This includes integrating farm system practices, such as nutrient budgeting, grazing management, and excreta handling, with well-placed edge-of-field interventions to intercept losses before they reach waterways. Achieving meaningful and sustained reductions will depend on aligning actions with farm profitability, supported by policy frameworks that combine incentives, regulation, and continual improvement through robust monitoring.
